# ExGUtils: A Python Package for Statistical Analysis With the ex-Gaussian Probability Density

**DOI:** 10.3389/fpsyg.2018.00612

**Published:** 2018-05-01

**Authors:** Carmen Moret-Tatay, Daniel Gamermann, Esperanza Navarro-Pardo, Pedro Fernández de Córdoba Castellá

**Affiliations:** ^1^Department of Neuropsychology, Methodology, Basic and Social Psychology, Faculty of Psychology, Universidad Católica de Valencia San Vicente Mártir, Valencia, Spain; ^2^Instituto de Física, Universidade Federal do Rio Grande do Sul (UFRGS), Porto Alegre, Brazil; ^3^Department of Developmental and Educational Psychology, Faculty of Psychology, Universitat de Valencia, Valencia, Spain; ^4^Grupo de Modelización Interdisciplinar, Instituto Universitario de Matemática Pura y Aplicada, InterTech, Universitat Politècnica de València, Valencia, Spain

**Keywords:** response times, response components, python, ex-Gaussian fit, significance testing

## Abstract

The study of reaction times and their underlying cognitive processes is an important field in Psychology. Reaction times are often modeled through the ex-Gaussian distribution, because it provides a good fit to multiple empirical data. The complexity of this distribution makes the use of computational tools an essential element. Therefore, there is a strong need for efficient and versatile computational tools for the research in this area. In this manuscript we discuss some mathematical details of the ex-Gaussian distribution and apply the ExGUtils package, a set of functions and numerical tools, programmed for python, developed for numerical analysis of data involving the ex-Gaussian probability density. In order to validate the package, we present an extensive analysis of fits obtained with it, discuss advantages and differences between the least squares and maximum likelihood methods and quantitatively evaluate the goodness of the obtained fits (which is usually an overlooked point in most literature in the area). The analysis done allows one to identify outliers in the empirical datasets and criteriously determine if there is a need for data trimming and at which points it should be done.

## 1. Introduction

The reaction time (RT) has become one of the most popular dependent variables in cognitive psychology. Over the last few decades, much research has been carried out on problems focusing exclusively on success or fail in trials during the performance of a task, emphasizing the importance of RT variables and their relationship to underlying cognitive processes (Sternberg, 1966; Wickelgren, 1977; McVay and Kane, 2012; Ratcliff et al., 2012). However, RT has a potential disadvantage: its skewed distribution. One should keep in mind that in order to perform data analysis, it is preferable that the data follow a known distribution. If the distribution is not symmetrical, it is possible to carry out some data transformation techniques (e.g., the Tukey scale for correcting skewness distribution), or to apply some trimming techniques, but with these techniques, statistics may be altered (in other words a high concentration of cases in a given range may be favored and as a result, statistics can appear biased). Moreover, transformations can affect the absolute value of the data or modify the relative distances between data. When conducting trimming it is not easy to distinguish noisy data from valid information, or in other words, to set the limits between outliers and extreme data (Heathcote et al., 1991). Whether we include or exclude outliers often depends on the reason why they might occur, dealing with the decision to classify them as variability in the measurement or as an experimental error. Another option, for the analysis of skewed data, is to characterize them with a known skewed distribution. This procedure allows one to determine the probability of an event based on the statistical model used to fit the data. A common problem with this approach is to estimate the parameters that characterize the distribution. In practice, when one wants to find out the probability for an event numerically, a quantified probability distribution is required.

Going back to the point on characterizing data with a specific distribution, there is one distribution that has been widely employed in the literature when fitting RT data: the exponentially modified Gaussian distribution (West, 1999; Leth-Steensen et al., 2000; West and Alain, 2000; Balota et al., 2004; Hervey et al., 2006; Epstein et al., 2011; Gooch et al., 2012; Navarro-Pardo et al., 2013). This distribution is characterized by three parameters, μ, σ and τ. The first and second parameters (μ and σ), correspond to the average and standard deviation of the Gaussian component, while the third parameter (τ) is the decay rate of the exponential component. This distribution provides good fits to multiple empirical RT distributions (Luce, 1986; Lacouture and Cousineau, 2008; Ratcliff and McKoon, 2008), however there are currently no published statistical tables available for significance testing with this distribution, though there are softwares like S-PLUS (Heathcote, 2004) or PASTIS (Cousineau and Larochelle, 1997) and programming language packages available for R, MatLab or Methematica.

In this article we present a package, developed in Python, for performing statistical and numerical analysis of data involving the ex-Gaussian function. Python is a high-level interpreted language. Python and R are undoubtedly two of the most widespread languages, as both are practical options for building data models with a lot of community support. However, the literature seems to be rather scarce in terms of computations with the ex-Gaussian function in Python. The package presented here is called ExGUtils (from ex-Gaussian Utilities), it comprises functions for different numerical analysis, many of them specific for the ex-Gaussian probability density.

The article is organized as follows: in the next section we present the ex-Gaussian distribution, its parameters and a different way in which the distribution can be parameterized. Following this, we discuss two fitting procedures usually adopted to fit probability distributions: the least squares and the maximum likelihood. In the third section we present the ExGUtils module and we apply it in order to fit experimental data, evaluate the goodness of the fits and discuss the main differences in the two fitting methods. In the last section we present a brief overview.

## 2. The ex-Gaussian distribution and its probability density

Given a randomly distributed *X* variable that can assume values between minus infinity and plus infinity with probability density given by the gaussian distribution,

(1) $$g(x)=\frac{1}{\sigma \sqrt{2\pi }}\exp\left(-\frac{1}{2}{\left(\frac{x-\mu }{\sigma }\right)}^{2}\right),$$

and a second random *Y* variable that can assume values between zero and plus infinity with probability density given by an exponential distribution,

(2) $$h(x)=\frac{1}{\tau }e^{-\frac{x}{\tau }},$$

let's define the *Z* variable as the sum of the two previous random variables: *Z* = *X* + *Y*.

The gaussian distribution has average μ and standard deviation σ, while the average and standard deviation of the *Y* variable will be both equal to τ. The *Z* variable will also be a random variable, whose average will be given by the sum of the averages of *X* and *Y* and whose variance will be equal to the sum of the variances of *X* and *Y*:

(3) M=μ+τ

(4) $$S^{2}=\sigma^{2}+\tau^{2}$$

Defined as such, the variable *Z* has a probability density with the form (Grushka, 1972):

(5) $$f(x)=\frac{1}{2\tau }\exp\left(\frac{1}{2\tau }\left(2\mu +\frac{\sigma^{2}}{\tau }-2x\right)\right)\text{erfc}\left(\frac{\mu +\frac{\sigma^{2}}{\tau }-x}{\sqrt{2}\sigma }\right)$$

which receives the name of ex-Gaussian distribution (from exponential modified gaussian distribution). The erfc function is the complementary error function. One must be careful, for μ and σ are NOT the average and standard deviation for the ex-Gaussian distribution, instead the average and variance of the ex-Gaussian distribution is given by Equations (3)–(4): *M* = μ + τ and *S*^2^ = σ^2^+τ^2^. On the other hand, a calculation of the skewness of this distribution results in:

(6) $$K=\int_{-\infty }^{\infty }{\left(\frac{x-M}{S}\right)}^{3}f(x)\text{d}x=\frac{2\tau^{3}}{{\left(\sigma^{2}+\tau^{2}\right)}^{\frac{3}{2}}},$$

While the gaussian distribution has null skewness, the skewness of the exponential distribution is exactly equal to two. As a result the skewness of the ex-Gaussian has an upper bound equal to two in the limit σ ≪ τ (when the exponential component dominates) and a lower bound equal to zero in the limit σ ≫ τ (when the gaussian component dominates).

Let's parameterize the ex-Gaussian distribution in terms of its average *M*, standard deviation *S* and a new skewness parameter $\lambda =\sqrt[{3}]{\frac{K}{2}}$. Defined in this way, the λ parameter can have values between 0 and 1. Now, defining the standard coordinate *z* ($z=\frac{x-M}{S}$) one can have the ex-Gaussian distribution normalized for average 0 and standard deviation 1 in terms of a single parameter, its asymmetry λ:

(7) $$f_{\lambda }(z)=\frac{1}{2\lambda }\exp\left(\frac{1}{2\lambda^{2}}(-2z\lambda -3\lambda^{2}+1)\right)\text{erfc}\left(\frac{-z+\frac{1}{\lambda }-2\lambda }{\sqrt{2}\sqrt{1-\lambda^{2}}}\right).$$

in this case, in terms of λ, the parameters μ, σ and τ are given by:

(8) μ=−λ

(9) $$\sigma =\sqrt{1-\lambda^{2}}$$

(10) τ=λ.

Thus, the ex-gaussian represents a family of distributions that can be parametrized in terms of their assymmetry. Ranging from the exponential (maximum assymmetry in the limit when λ = 1) to a gaussian (symmetrical distribution in the limit when λ = 0).

**Figure 1:**
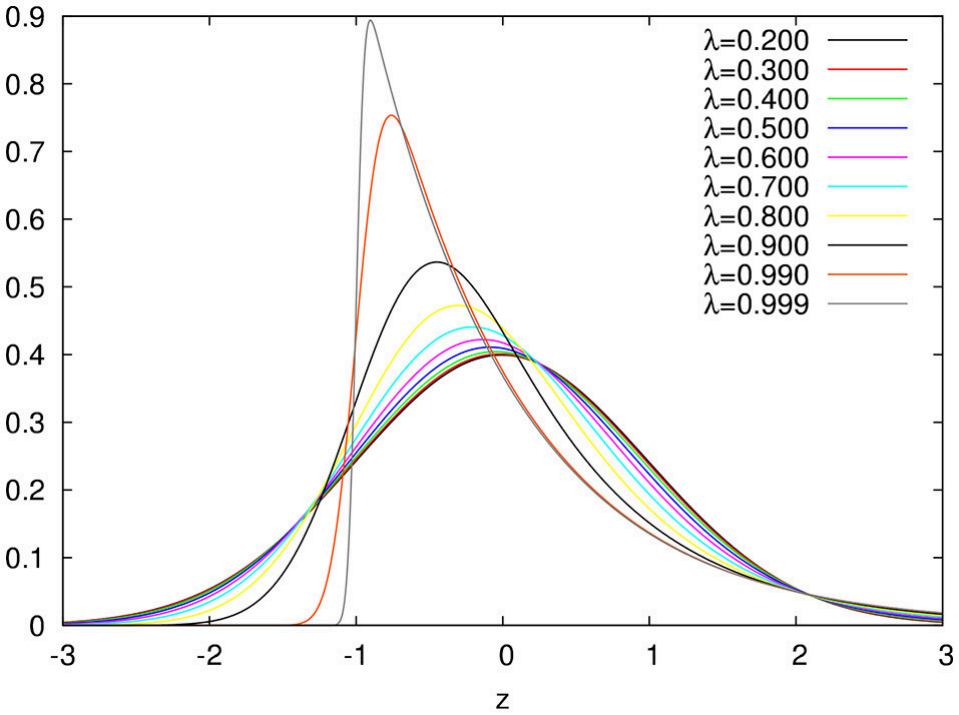
ex-Gaussian distributions for different values of the λ asymmetry parameter.

**Figure 2:**
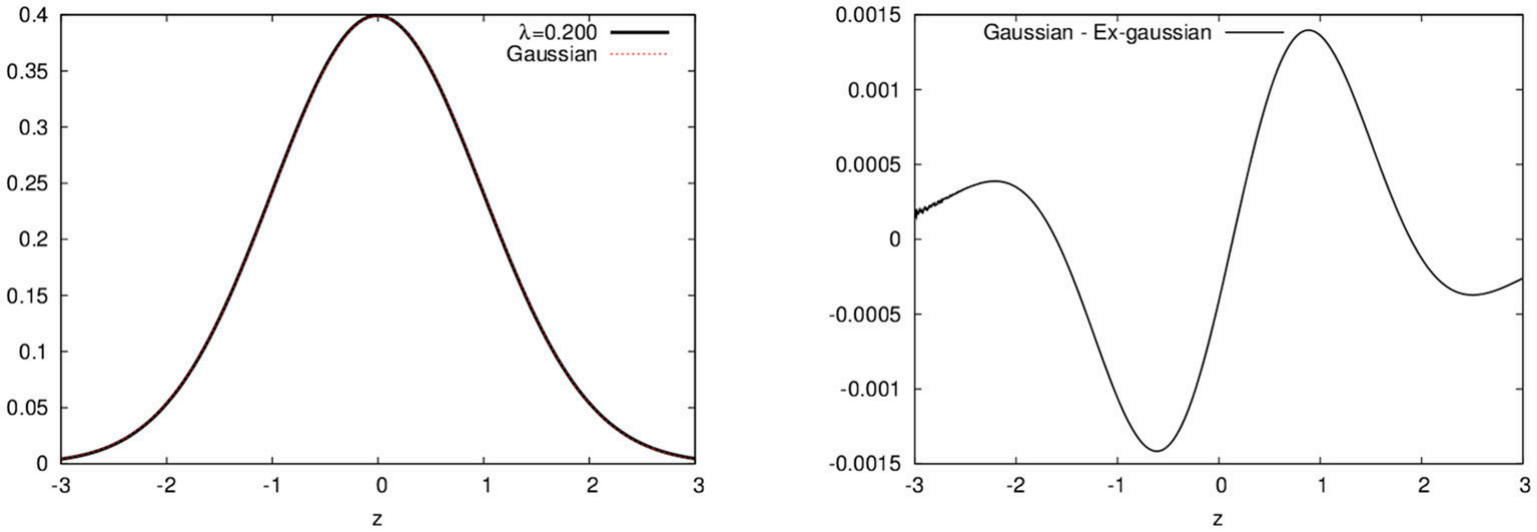
Differences between the ex-Gaussian distribution with λ = 0.2 and the gaussian distribution. Both curves plotted on the left and the difference on the right (note this difference is less than 1%).

In Figure 1, we show plots for the ex-Gaussian function for different values of the parameter λ. We should note that for very small values of λ (less than around 0.2), the ex-Gaussian is almost identical to the gaussian function (see Figure 2)^1^.

Given a probability density, an important function that can be calculated from it is its cumulative distribution (its left tail), which is the result of the integral

(11) $$F(z)=\int_{-\infty }^{z}f(x)\text{d}x.$$

The importance of this function is that given the cumulative distribution one is able to calculate the probability of an event. For the ex-gaussian, the expression for its cumulative distribution is given by:

(12) $$\begin{array}{l} F(x)=\frac{1}{2}\text{erfc}\left(-\frac{x-\mu }{\sqrt{2}\sigma }\right)-\frac{1}{2}\exp\left(\frac{\sigma^{2}}{\tau^{2}}-\frac{x-\mu }{\tau }\right)\\ \text{ erfc}\left(-\frac{\frac{x-\mu }{\sigma }-\frac{\sigma }{\tau }}{\sqrt{2}}\right) \end{array}$$

Let's also define *z*_α_, the value of *z* for which the right tail of the distribution has an area equal to α:

(13) $$\alpha =\int_{z_{\alpha }}^{\infty }f(x)\text{d}x.$$

(14) $$1-F(z_{\alpha })=\alpha$$

so, solving the Equation (14), one is able to obtain the value of *z*_α_ for any given α.

## 3. Fitting the probability distribution

We are interested in the following problem: given a dataset, to estimate the parameters μ, σ and τ that, plugged into Equation (5), best fit the data.

We must now define what it means to best fit the data. Different approaches here will result in different values for the parameters. The most trivial approach would be to say that the best parameters are those that result in the fitted ex-Gaussian distribution with the same statistical parameters: average (*M*), standard deviation (*S*) and asymmetry (*K* or λ). So, one can take the dataset, calculate *M*, *S*, and *K* and use the relations between them and the parameters μ, σ and τ:

(15) M=μ+τ

(16) $$S=\sqrt{\sigma^{2}+\tau^{2}}$$

(17) $$\lambda =\sqrt[{3}]{\frac{K}{2}}=\frac{\tau }{\sqrt{\sigma^{2}+\tau^{2}}}$$

(18) μ=M−Sλ

(19) $$\sigma =S\sqrt{1-\lambda^{2}}$$

(20) τ=Sλ

This method of evaluating the parameters from the statistic (*momenta*) is know as the method of the moments as is usually the worst possible approach given the resulting bias. For instance, in some experiments, one finds the *K* parameter bigger than 2 (or λ > 1) and from Equation (17) one sees that, in order to have *K* > 2, σ cannot be a real number.

Another approach is to find the parameters that minimize the sum of the squared differences between the observed distribution and the theoretical one (least squares). In order to do that, one must, from the dataset, construct its distribution (a histogram), which requires some parametrization (dividing the whole range of observations in fixed intervals). Since a potentially arbitrary choice is made here, the results might be dependent on this choice. When analyzing data, we will study this dependency and come back to this point.

The last approach we will study is the maximum likelihood method. The function in Equation (5) is a continuous probability distribution for a random variable, which means that *f*(*x*)*dx* can be interpreted as the probability that a observation of the random variable will have the *x* value (with the infinitesimal uncertainty *dx*). So, given a set of *N* observations of the random variable, {*x*_*i*_}, with *i* = 1, 2, …, *N*, the likelihood L is defined as the probability of such a set, given by:

(21) $$L=\prod_{i=1}^{N}f(x_{i};\mu ,\sigma ,\tau )$$

(22) $$\ln L=\sum_{i=1}^{N}\ln(f(x_{i};\mu ,\sigma ,\tau ))$$

The maximum likelihood method consists in finding the parameters μ, σ and τ that maximize the likelihood L (or its logarithm^2^ lnL). Note that in this approach, one directly uses the observations (data) without the need of any parametrization (histogram).

In both approaches, least squares and maximum likelihood, one has to find the extreme (maximum or minimum) of a function. The numerical algorithm implemented for this purpose is the steepest descent/ascent (descent for the minimum and ascent for the maximum). The algorithm consists in interactively changing the parameters of the function by amounts given by the gradient of the function in the parameter space until the gradient falls to zero (to a certain precision). There are other optimization methods, like the simplex (Van Zandt, 2000; Cousineau et al., 2004), which also iteratively updates the parameters (in the case of the simplex without the need to compute the gradients). We chose to implement steepest ascent in order to gain in efficiency: since one is able to evaluate the gradients, this greedy algorithm should converge faster than the sample techniques used by simplex. But in any case, both algorithms (steepest descent and simplex) should give the same results, since both search the same maximum or minimum.

## 4. The ExGUtils module

ExGUtils is a python package with two modules in its 3.0 version: one purely programmed in python (pyexg) and the other programmed in C (uts). The advantage of having the functions programmed in C is speed, stability and numerical precision.

As mentioned, the package has two modules: pyexg and uts. The first one comprises all functions with source code programed in python, some of which depend on the numpy, scipy and random python packages. On the other hand, the module uts contains functions with source code programmed in C. In Table 1 one can find a complete list of all functions contained in both modules and the ones particular to each one. The source distribution of the ExGUtils module comes with a manual which explains in more detail and with examples the functions.

**Table 1:** Functions present in the package modules.

**Module**	**Function**	**Brief description**
Present	drand	Returns a random number with homogeneous distribution between 0 and 1
in	exp_rvs	Returns a random number with exponential distribution between 0 and infinity
both	gauss_rvs	Returns a random number with gaussian distribution between minus infinity and infinity
modules	exg_rvs	Returns a random number with ex-Gaussian distribution between minus infinity and infinity
	gauss_pdf	Evaluates the gaussian distribution at a given point
	gauss_cdf	Evaluates the gaussian cumulative distribution at a given point
	exg_pdf	Evaluates the ex-Gaussian distribution at a given point
	exg_cdf	Evaluates the ex-Gaussian cumulative distribution at a given point
	exg_lamb_pdf	Evaluates the ex-Gaussian distribution parameterized in terms of its asymmetry at a given point
	exg_lamb_cdf	Evaluates the ex-Gaussian cumulative distribution parameterized in terms of its asymmetry at a given point
	pars_to_stats	Given the parameters μ, σ and τ, evaluates the corresponding statistics *M*, *S*, and *K*
	stats_to_pars	Given the statistics *M*, *S* and *K*, evaluates the corresponding parameters μ, σ and τ
	histogram	Given a set of observations, produces an histogram
	stats	Given a set of observations, returns the statistics *M*, *S*, and *K*
	stats_his	Given a set of observations, presented as a histogram, returns the statistics *M*, *S*, and *K*
	correlation	Given a set of points, returns the linear correlation coefficient for the points
	minsquare	Given a set of points, fits a polynomial to the data using the least square method
	exgLKHD	Evaluates the likelihood and its gradient in the parameter space for a dataset in a given point of the parameter space
	maxLKHD	Evaluates the parameters μ, σ and τ that maximize the likelihood for a given dataset
	exgSQR	Evaluates the sum of squared differences and its gradient in the parameter space for an histogram in a given point of the parameter space
	minSQR	Evaluates the parameters μ, σ and τ that minimize the sum of squared differences for a given histogram
Only	int_points_gauss	Creates a point partition of an interval for evaluating a
in		gaussian integral
uts	intsum	Evaluates the gaussian integral for a function calculated at the points in a gaussian partition
Only	zero	Finds the zero of an equation
in	ANOVA	Performs an ANOVA test
pyexg	integral	Evaluates an integral

*In python type help(FUNC) (where FUNC should be the name of a given function), in order to obtain the list of arguments that each function should receive and in which order*.

## 5. Applications

We use here the ExGUtils package in order to analyze data from the experiment in Navarro-Pardo et al. (2013). From this work, we analyse the datasets obtained for the reaction times of different groups of people in recognizing different sets of words in two possible experiments (yes/no and go/nogo). In the Appendix B we briefly explain the datasets analyzed here (which are provided as Supplementary Material for download).

In our analysis, first each dataset is fitted to the ex-Gaussian distribution through the three different approaches aforementioned:

- moments → Estimating the parameters through the sample statistics Equations (18–20).
- minSQR → Estimation through least square method, using as initial point in the steepest descent algorithm the μ, σ and τ obtained from the method of moments above^3^.
- maxLKHD → Estimation through maximum likelihood method, using as initial point in the steepest ascent algorithm the μ, σ and τ obtained from the method of moments^3^.

In Table 2, one can see the estimated parameters and the corresponding statistics for the different experiments. From the table, one sees that in the case of the experiments performed with young people, the value of the skewness, *K*, is bigger than two. This happens because of a few atypical measurements far beyond the bulk of the distribution. In fact, many researches opt for trimming extreme data, by “arbitrarly” choosing a cutoff and removing data points beyond this cutoff. One must, though, be careful for the ex-Gaussian distribution does have a long right tail, so we suggest a more criterious procedure:

**Table 2:** Parameters and statistics obtained with the three fitting methods.

**Experiment**	**Moments**						**minSQR**						**maxLKHD**					
	***M***	***S***	***K***	**μ**	**σ**	**τ**	***M***	***S***	***K***	**μ**	**σ**	**τ**	***M***	***S***	***K***	**μ**	**σ**	**τ**
elder_gng	831.14	318.95	1.75	526.06	93.02	305.08	841.45	334.97	1.85	515.16	75.76	326.29	831.14	318.39	1.79	524.49	85.67	306.65
elder_hfgng	798.55	310.00	1.94	491.52	42.78	307.03	803.42	308.32	1.82	504.66	76.18	298.76	796.63	312.32	1.90	489.39	56.11	307.24
elder_hfyn	826.15	278.61	1.62	566.56	101.16	259.59	810.03	256.41	1.68	568.03	84.74	242.00	826.15	275.10	1.75	563.06	80.40	263.08
elder_lfgng	863.73	324.53	1.60	562.65	121.14	301.08	887.69	371.88	1.90	522.22	68.76	365.46	863.73	339.88	1.87	531.43	71.38	332.30
elder_lfyn	884.53	315.93	1.59	591.97	119.27	292.55	882.16	310.04	1.77	584.33	86.12	297.84	884.53	308.13	1.62	597.54	112.18	286.98
elder_pseudo	1189.64	416.92	0.88	872.59	270.73	317.05	1233.90	518.22	1.79	734.21	137.33	499.69	1189.64	447.89	1.60	773.74	166.24	415.90
elder_yn	854.88	298.93	1.63	575.78	107.04	279.11	846.94	286.75	1.75	572.77	84.01	274.17	854.88	292.72	1.68	578.59	96.69	276.29
young_gng	597.90	169.90	2.71	–	–	–	590.36	142.94	1.66	455.98	48.71	134.38	597.90	154.25	1.72	451.09	47.33	146.81
young_hfgng	562.94	141.88	3.04	–	–	–	555.47	115.19	1.54	449.84	45.93	105.63	562.94	126.30	1.65	444.47	43.77	118.47
young_hfyn	621.16	176.99	3.88	–	–	–	610.51	128.15	1.53	493.32	51.86	117.19	621.16	148.38	1.65	482.08	51.70	139.08
young_lf	632.96	187.61	2.46	–	–	–	625.36	161.43	1.65	473.80	55.61	151.55	632.96	173.23	1.71	468.51	54.43	164.45
young_lfgng	632.96	187.61	2.46	–	–	–	625.36	161.43	1.65	473.80	55.61	151.55	632.96	173.23	1.71	468.51	54.43	164.45
young_lfyn	668.57	184.88	2.10	–	–	–	660.23	165.16	1.60	507.06	61.77	153.18	668.58	176.56	1.70	501.24	56.31	167.34
young_pseudo	722.53	190.36	2.37	–	–	–	718.60	175.25	1.64	554.66	61.92	163.94	722.53	180.58	1.68	552.03	59.47	170.50
young_yn	644.37	182.41	2.90	–	–	–	635.20	148.99	1.62	496.41	54.18	138.78	644.37	164.31	1.69	488.96	53.34	155.42

*For each one of the three procedures, we present the obtained parameters and the correspondent statistics. Note that for the method of the moments, the statistics is directly obtained from the dataset and the parameters are evaluated based on this statistic from Equations (18–20), while the other methods obtain the parameters from the dataset and the statistics presented in the table is evaluated based on these parameters from the expressions in eqs. Some conditions were not estimated because of a few atypical measurements far beyond the bulk of the distribution (K larger than 2). (15-17)*.

Having the tools developed in ExGUtils, one can use the parameters obtained in the fitting procedures (either minSQR or maxLKHD) in order to estimate a point beyond which one should find no more than, let's say, 0.1% of the distribution. In the Appendix A (Supplementary Material), the Listing 1 shows a quick python command line in order to estimate this point in the case of the young_gng experiment. The result informs us that, in principle, one should not expect to have more than 0.1% measurements of reaction times bigger than 1472.84 ms if the parameters of the distribution are the ones adjusted by maxLKHD for the young_gng empirical data. In fact, in this experiment, one has 2396 measurements of reaction times, from those, 8 are bigger than 1472.8 ms (0.33%). If one now calculates the statistics for the data, removing these 8 outliers, one obtains:

$$\begin{array}{l} \text{ moments}:M=593.80\;S=154.30\;K=1.91\;\mu \;=\;441.82\\ \;\sigma =26.67\;\tau \;=\;151.98\\ \text{ minSQR}:M=590.11\;S=142.44\;K=1.67\;\mu \;=\;455.96\\ \;\sigma \;=47.89\;\tau \;=\;134.14\\ \text{maxLKHD}:M=593.80\;S=148.44\;K=1.69\;\mu \;=\;453.52\\ \;\sigma =48.52\;\tau \;=\;140.29 \end{array}$$

**Figure 3:**
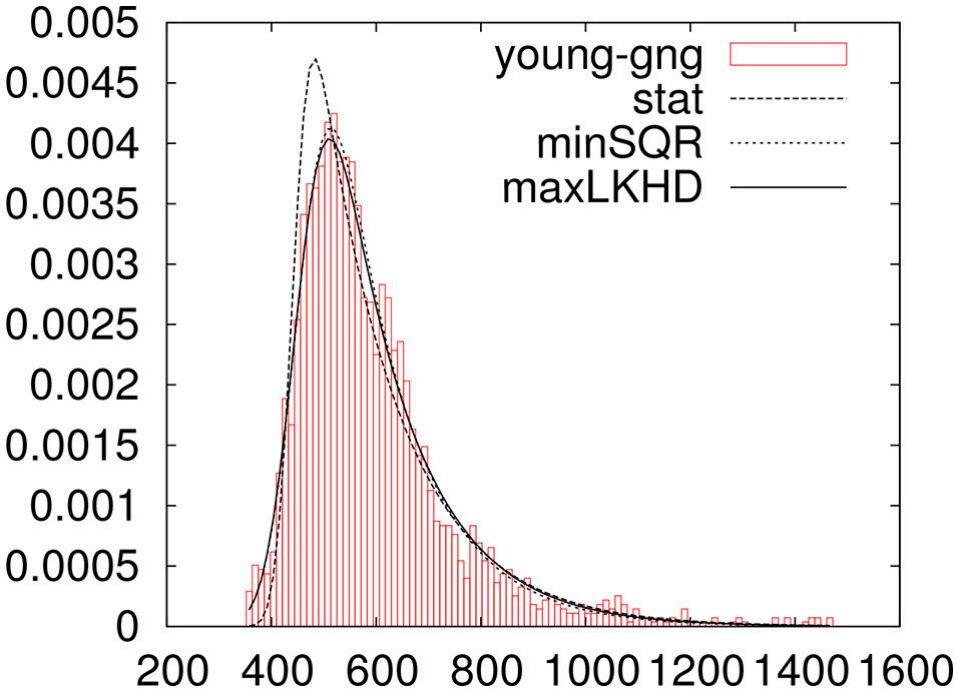
Data for the young_gng experiment trimmed for outliers with three fitted ex-Gaussians.

In Figure 3 one can see the histogram of data plotted along with three ex-Gaussians resulting from the above parameters.

Now, one might ask, having these different fits for the same experiment, how to decide which one is the best? Accepting the parameters of a fit is the same as accepting the null hypothesis that the data measurements come from a population with an ex-Gaussian distribution with the parameters given by the ones obtained from the fit. In Clauset et al. (2009) the authors suggest a procedure in order to estimate a *p*-value for this hypothesis when the distribution is a power-law. One can generalize the procedure for any probability distribution, like the ex-Gaussian, for example:

- Take a measure that quantifies the distance between the data and the fitted theoretical distribution. One could use ln L or χ^2^, but, as our fitting procedures maximize or minimize these measures, as the authors in Clauset et al. (2009) suggest, in order to avoid any possible bias, we evaluate the Kolmogorov-Smirnov statistic, which can be calculated for reaction-time data without the need of any parametrization.
- Randomly generate many data samples of the same size as the empirical one using the theoretical distribution with the parameters obtained from the fit to the empirical data.
- Fit each randomly generated data sample to the theoretical distribution using the same fit procedure used in the case of the empirical data.
- Evaluate the Kolmogorov-Smirnov statistic between the random sample and its fitted theoretical distribution.

Following this procedure, one can evaluate the probability that a random data sample, obtained from the fitted distribution, has a bigger distance to the theoretical curve than the distance between the empirical data and its fitted distribution. If this probability is higher than the confidence level one is willing to work with, one can accept the null hypothesis knowing that the probability that one is committing a type I error if one rejects the null hypothesis is *p*.

In the Appendix A (Supplementary Material) we provide listings with the implementation, in python via the ExGUtils package, of the functions that evaluate this *p* probability and the Kolmogorov-Smirnov statistic. In Table 3 we provide the values of *p* obtained for the experiments, using minSQR and maxLKHD approaches (p1 and p2, respectively).

**Table 3:** Probabilities p1 and p2 for the fits.

**Experiment**	**minSQR**		**maxLKHD**	
	**KS**	**p2 ($\bar{\text{KS}}\pm sd$)**	**KS**	**p1 ($\bar{\text{KS}}\pm sd$)**
elder_gng	64.52	0.001 (29.47 ± 8.12)	38.89	0.096 (29.96 ± 12.54)
elder_hfgng	44.32	0.001 (20.85 ± 5.73)	49.61	0.003 (21.33 ± 5.86)
elder_hfyn	34.10	0.019 (20.10 ± 5.35)	35.30	0.021 (20.44 ± 7.49)
elder_lfgng	42.83	0.005 (21.73 ± 5.98)	31.70	0.043 (20.96 ± 5.94)
elder_lfyn	17.25	0.634 (19.76 ± 5.18)	29.00	0.028 (19.15 ± 5.63)
elder_pseudo	62.79	0.000 (26.12 ± 6.81)	53.10	0.009 (25.69 ± 10.41)
elder_yn	32.87	0.258 (28.77 ± 7.42)	62.72	0.012 (29.00 ± 14.16)
young_gng	35.92	0.136 (28.60 ± 7.39)	69.38	0.003 (28.66 ± 8.36)
young_hfgng	21.33	0.305 (19.70 ± 4.99)	34.11	0.016 (20.13 ± 6.16)
young_hfyn	29.75	0.049 (19.59 ± 5.04)	45.20	0.009 (19.83 ± 7.03)
young_lf	22.06	0.318 (20.39 ± 5.81)	37.78	0.015 (20.67 ± 7.82)
young_lfgng	22.06	0.299 (20.08 ± 5.25)	37.78	0.012 (20.27 ± 6.52)
young_lfyn	23.62	0.182 (19.66 ± 5.03)	17.66	0.542 (19.56 ± 7.43)
young_pseudo	20.35	0.867 (27.86 ± 7.20)	28.48	0.386 (28.44 ± 10.87)
young_yn	38.34	0.097 (28.07 ± 7.03)	54.20	0.003 (28.13 ± 8.66)

*KS is the Kolmogorov-Smirnov statistic calculated between the data and its fitted ex-Gaussian. In columns p1 and p2, one finds the probabilities that a randomly generated dataset has a bigger KS statistic than the empirical data. In parenthesis, the average KS statistic and standard deviation for the generated random samples*.

We can see that there are some discrepancies in Table 3. Sometimes minSQR seems to perform better, sometimes maxLKHD. One might now remember that the minSQR method depends on a parametrization of the data. In order to perform the fit, one needs to construct a histogram of the data, and there is an arbitrary choice in the number of intervals one divides the data into. In the fits performed till now, this number is set to be the default in the histogram function of the ExGUtils package, namely two times the square root of the number of measurements in the data.

**Figure 4:**
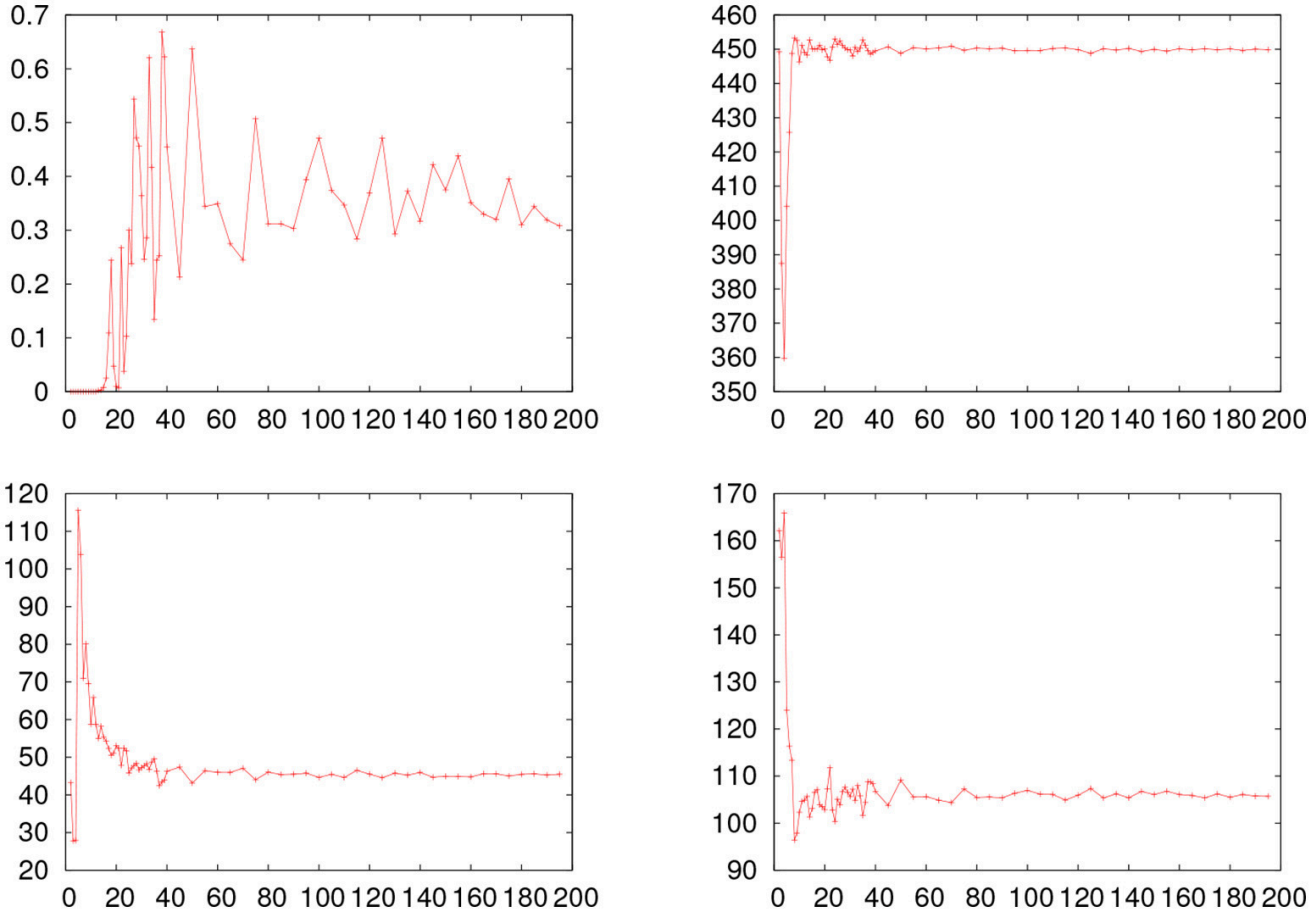
Fitting results obtained with the minSQR method varying the number of intervals in the histogram for the young_hfgng experiment. The horizontal line shows the value obtained with the maxLKHD method. **(Upper left)** Evolution of the *p* probability. **(Upper right)** Evolution of μ. **(Bottom left)** Evolution of σ. **(Bottom right)** Evolution of τ.

In order to study the effect of the number of intervals in the values for the parameters and of p2, we performed the procedure of fitting the data through minSQR after constructing the histogram with different number of intervals. In Figure 4 we show the evolution of the p2 probability, along with the values for μ, σ, and τ obtained by minSQR for the histograms constructed with a different number of intervals for the young_hfgng experiment.

From the figure one sees that while the number of intervals is unreasonably small compared to the size of the empirical dataset, the values for the fitted ex-Gaussian parameters fluctuate, while the *p* probability is very small, but, once the number of intervals reaches a reasonable value, around 40, the values for the parameters stabilize and the value of *p* also gets more stable. So the question remains, why the values for the probability obtained with maxLKHD method is so small in the case of this experiment? The fact is that the likelihood of the dataset is very sensible to outliers. For the value of the probability [*f*(*x*) in Equation 5] gets very small for the extreme values. Therefore, in these cases, it might be reasonable to make some criterious data trimming. So we proceed as follows: Given a dataset, we first perform a pre-fitting by maxLKHD. Using the parameters obtained in this fit, we estimate the points where the distribution has a left and right tails of 0.1% and remove measurements beyond these points. With the trimmed dataset, removed of outliers, we perform fits again and evaluate the p1 and p2 probabilities. In Table 4, we show the results for this new round of fitting and probability evaluations. In more than half of the experiments where one could see a big discrepancy between p1 and p2 in Table 3, the trimmed data do show better results. For some datasets, the trimming had no impact on the discrepancy. In any case, one might wonder about the impact of the trimming in the obtained parameters. Therefore, in Table 5, we show the results obtained with different trimming criteria.

**Table 4:** The p1 and p2 probabilities for the fits.

**Experiment**	***N***	**N′ (%)**	**minSQR**		**maxLKHD**	
			**KS**	**p2 ($\bar{\text{KS}}\pm sd$)**	**KS**	**p1 ($\bar{\text{KS}}\pm sd$)**
elder_gng	2,348	2 (0.09)	66.58	0.000 (28.92 ± 7.32)	50.24	0.040 (30.98 ± 17.55)
elder_hfgng	1,174	8 (0.68)	34.20	0.040 (20.67 ± 5.70)	32.64	0.010 (20.66 ± 5.83)
elder_hfyn	1,175	2 (0.17)	32.09	0.040 (20.01 ± 4.86)	24.76	0.090 (19.22 ± 6.69)
elder_lfgng	1,174	1 (0.09)	43.49	0.000 (21.47 ± 5.83)	33.22	0.030 (20.57 ± 6.90)
elder_lfyn	1,139	4 (0.35)	19.97	0.550 (20.55 ± 6.37)	19.71	0.620 (19.97 ± 6.11)
elder_pseudo	1,910	5 (0.26)	57.26	0.000 (26.91 ± 6.64)	57.11	0.010 (26.61 ± 10.06)
elder_yn	2,314	5 (0.22)	36.83	0.240 (28.57 ± 7.46)	29.72	0.230 (30.54 ± 14.33)
young_gng	2,396	10 (0.42)	38.93	0.250 (27.82 ± 6.32)	43.11	0.020 (30.19 ± 17.07)
young_hfgng	1,200	8 (0.67)	23.28	0.780 (19.25 ± 4.39)	17.82	0.430 (18.07 ± 4.13)
young_hfyn	1,180	9 (0.76)	27.97	0.050 (19.68 ± 4.91)	28.93	0.010 (20.74 ± 7.71)
young_lf	1,196	5 (0.42)	25.11	0.310 (20.09 ± 5.21)	25.32	0.020 (19.69 ± 4.29)
young_lfgng	1,196	5 (0.42)	25.11	0.280 (20.51 ± 5.08)	25.32	0.080 (20.55 ± 5.05)
young_lfyn	1,132	3 (0.27)	25.20	0.230 (19.42 ± 5.40)	16.60	0.780 (20.72 ± 8.53)
young_pseudo	2,326	10 (0.43)	23.33	0.940 (27.59 ± 7.05)	25.85	0.870 (28.45 ± 12.48)
young_yn	2,312	12 (0.52)	46.10	0.130 (27.80 ± 7.87)	28.58	0.210 (31.21 ± 19.74)

*KS is the Kolmogorov-Smirnov statistic calculated between the data and its fitted ex-Gaussian. N is the number of data points in each empirical dataset, N′ in the number of points removed by the trimming and in brackets next to it its proportion in relation to the total data. In columns p1 and p2, one finds the probabilities that a randomly generated dataset has a bigger KS statistic than the empirical data. In parenthesis, the average KS statistic and standard deviation for the generated random samples*.

**Table 5:** Results for different trimming on the data.

**Experiment**	**%**	**minSQR**				**maxLKHD**			
		**μ**	**σ**	**τ**	**p2**	**μ**	**σ**	**τ**	**p1**
elder_gng	0.1	513.52	73.00	329.54	0.001	518.71	75.02	313.04	0.026
elder_gng	0.5	516.62	76.61	319.50	0.002	521.83	70.31	299.00	0.011
elder_gng	1.0	516.04	76.80	317.93	0.000	523.84	66.32	291.17	0.014
elder_hfgng	0.1	509.10	84.96	285.05	0.043	504.96	65.26	297.06	0.012
elder_hfgng	0.5	509.39	89.51	277.28	0.020	511.19	65.09	277.33	0.020
elder_hfgng	1.0	508.40	83.49	279.35	0.016	512.79	59.89	272.67	0.005
elder_hfyn	0.1	564.82	82.19	246.63	0.052	558.93	71.17	266.45	0.148
elder_hfyn	0.5	565.70	83.88	242.73	0.036	559.98	68.60	261.73	0.143
elder_hfyn	1.0	566.73	87.05	235.38	0.006	561.88	65.77	255.95	0.094
elder_lfgng	0.1	521.64	62.39	368.34	0.006	530.64	68.95	333.51	0.041
elder_lfgng	0.5	523.29	67.46	359.50	0.006	530.25	60.81	329.35	0.011
elder_lfgng	1.0	523.37	67.70	356.20	0.002	533.09	59.45	318.33	0.008
elder_lfyn	0.1	583.03	84.58	301.15	0.562	581.72	76.56	305.56	0.577
elder_lfyn	0.5	584.32	86.07	296.15	0.524	584.60	78.19	296.28	0.329
elder_lfyn	1.0	586.72	85.93	287.48	0.470	589.73	77.85	278.47	0.027
elder_pseudo	0.1	735.04	133.55	498.90	0.001	755.81	134.79	436.48	0.012
elder_pseudo	0.5	733.65	135.57	499.00	0.001	754.68	132.25	438.02	0.017
elder_pseudo	1.0	732.54	135.87	498.14	0.000	752.31	124.65	442.19	0.014
elder_yn	0.1	572.16	81.99	275.26	0.251	567.87	73.30	288.63	0.280
elder_yn	0.5	573.64	84.34	270.01	0.373	570.72	72.30	278.01	0.378
elder_yn	1.0	573.82	84.87	266.60	0.246	573.48	72.59	268.80	0.159
young_gng	0.1	456.35	48.59	133.40	0.292	453.37	47.60	140.66	0.013
young_gng	0.5	456.95	47.02	132.15	0.177	456.29	43.54	134.00	0.167
young_gng	1.0	457.70	46.28	130.55	0.096	457.63	40.37	131.00	0.013
young_hfgng	0.1	449.79	45.31	105.15	0.707	448.42	44.89	109.02	0.565
young_hfgng	0.5	450.77	44.72	103.91	0.500	449.62	40.74	107.45	0.704
young_hfgng	1.0	451.94	44.75	101.09	0.208	451.50	37.51	103.23	0.226
young_hfyn	0.1	493.66	50.92	116.16	0.032	487.17	51.93	126.49	0.009
young_hfyn	0.5	494.62	50.74	114.27	0.054	488.97	51.00	122.73	0.025
young_hfyn	1.0	495.77	50.10	111.55	0.083	493.08	49.40	114.69	0.170
young_lf	0.1	473.36	54.44	151.84	0.287	471.09	54.85	157.76	0.037
young_lf	0.5	474.18	55.22	148.96	0.207	474.72	51.93	148.93	0.117
young_lf	1.0	475.03	54.10	147.35	0.067	475.22	45.69	148.46	0.019
young_lfgng	0.1	473.36	54.44	151.84	0.290	471.09	54.85	157.76	0.054
young_lfgng	0.5	474.18	55.22	148.96	0.201	474.72	51.93	148.93	0.119
young_lfgng	1.0	475.03	54.10	147.35	0.068	475.22	45.69	148.46	0.021
young_lfyn	0.1	508.16	61.53	151.83	0.228	503.17	57.27	162.27	0.776
young_lfyn	0.5	508.79	62.11	148.82	0.306	506.82	56.33	153.58	0.713
young_lfyn	1.0	508.92	59.52	148.67	0.278	508.72	51.89	151.43	0.545
young_pseudo	0.1	555.42	63.03	161.81	0.951	555.36	60.57	162.27	0.858
young_pseudo	0.5	556.11	63.54	159.16	0.364	556.92	57.17	158.77	0.194
young_pseudo	1.0	557.18	62.50	157.25	0.096	559.57	54.06	153.59	0.021
young_yn	0.1	497.56	54.59	136.65	0.141	492.23	53.69	146.70	0.144
young_yn	0.5	498.05	54.18	135.23	0.374	495.25	52.33	139.85	0.605
young_yn	1.0	498.17	53.86	134.10	0.556	496.97	50.70	136.71	0.494

*The column % indicates the amount of tail trimmed to the left and right of the data*.

Now, having the full picture, one can realize that some values of *p* are indeed small, indicating that either the ex-Gaussian distribution is not that good a model in order to fit the empirical results, or there is still some systematic error in the analysis of the experiments. Most of these empirical datasets where one sees very low values of *p* are with elderly people. These have the τ parameter much bigger than the σ which indicates a very asymmetric distribution with a long right tail. Indeed, a careful analysis of the histograms will show that the tail in these empirical distributions seems to be cut short at the extreme of the plots, so that the limit time in the experiment should be bigger than 2,500 ms in order to get the full distribution. One might argue that the trimming actually was removing data, but most of the removed points in the trimming of elderly data, was from the left tail and not from the right. This issue will result in the wrong evaluation of the KS statistics, since it assumes that one is dealing with the full distribution. This kind of analysis might guide better experimental designs.

## 6. Overview

The ex-Gaussian fit has turned into one of the preferable options when dealing with positive skewed distributions. This technique provides a good fit to multiple empirical data, such as reaction times (a popular variable in Psychology due to its sensibility to underlying cognitive processes). Thus, in this work we present a python package for statistical analysis of data involving this distribution.

This tool allows one to easily work with alternative strategies (fitting procedures) to some traditional analysis like trimming. This is an advantage given that an ex-Gaussian fit includes all data while trimming may result in biased statistics because of the cuts.

Moreover, this tool is programmed as Python modules, which allow the researcher to integrate them with any other Python resource available. They are also open-source and free software which allows one to develop new tools using these as building blocks.

## 7. Availability

ExGUtils may be downloaded from the Python Package index (https://pypi.python.org/pypi/ExGUtils/3.0) for free along with the source files and the manual with extended explanations on the functions and examples.

## Author contributions

CM-T participated in the conception, design, and interpretation of data, and in drafting the manuscript. DG participated in the design, and analysis and interpretation of data, and in drafting the manuscript. EN-P and PF participated in revising the manuscript.

### Conflict of interest statement

The authors declare that the research was conducted in the absence of any commercial or financial relationships that could be construed as a potential conflict of interest.

## Footnotes

^1^In this cases, the numerical evaluation of the ex-Gaussian distribution in Equation (5) becomes unstable and one can without loss (to a precision of around one part in a million) approximate the ex-Gaussian by a gaussian distribution.

^2^Note that, since the logarithm is an monotonically increasing function, the maximal argument will result in the maximum value of the function as well.

^3^In the cases where *K* was bigger than 2, the inicial parameters were calculated as if *K* = 1.9. Note that the final result of the search should not depend on the inicial search point if it starts close to the local maximum/minimum.

## References

[B1] Balota D. A. Cortese M. J. Sergent-Marshall S. D. Spieler D. H. Yap M. (2004). Visual word recognition of single-syllable words. J. Exp. Psychol. Gen. 133:283. 10.1037/0096-3445.133.2.283 15149254

[B2] Clauset A. Shalizi C. R. Newman M. E. J. (2009). Power-Law distributions in empirical data. SIAM Rev. 51, 661–703. 10.1137/070710111

[B3] Cousineau D. Brown S. Heathcote A. (2004). Fitting distributions using maximum likelihood: methods and packages. Behav. Res. Methods Instrum. Comput. 36, 742–756. 10.3758/BF03206555 15641420

[B4] Cousineau D. Larochelle S. (1997). Pastis: a program for curve and distribution analyses. Behav. Res. Methods Instrum. Comput. 29, 542–548. 10.3758/BF03210606

[B5] Epstein J. N. Langberg J. M. Rosen P. J. Graham A. Narad M. E. Antonini T. N.. (2011). Evidence for higher reaction time variability for children with adhd on a range of cognitive tasks including reward and event rate manipulations. Neuropsychology 25:427. 10.1037/a0022155 21463041 PMC3522094

[B6] Gooch D. Snowling M. J. Hulme C. (2012). Reaction time variability in children with adhd symptoms and/or dyslexia. Dev. Neuropsychol. 37, 453–472. 10.1080/87565641.2011.650809 22799763 PMC3413905

[B7] Grushka E. (1972). Characterization of exponentially modified gaussian peaks in chromatography. Anal. Chem. 44, 1733–1738. 10.1021/ac60319a011 22324584

[B8] Heathcote A. (2004). Fitting wald and ex-wald distributions to response time data: an example using functions for the s-plus package. Behav. Res. Methods Instrum. Comput. 36, 678–694. 10.3758/BF03206550 15641415

[B9] Heathcote A. Popiel S. J. Mewhort D. (1991). Analysis of response time distributions: an example using the stroop task. Psychol. Bull. 109:340.

[B10] Hervey A. S. Epstein J. N. Curry J. F. Tonev S. Eugene Arnold L. Keith Conners C.. (2006). Reaction time distribution analysis of neuropsychological performance in an adhd sample. Child Neuropsychol. 12, 125–140. 10.1080/09297040500499081 16754533

[B11] Lacouture Y. Cousineau D. (2008). How to use matlab to fit the ex-gaussian and other probability functions to a distribution of response times. Tutor. Quant. Methods Psychol. 4, 35–45. 10.20982/tqmp.04.1.p035

[B12] Leth-Steensen C. King Elbaz Z. Douglas V. I. (2000). Mean response times, variability, and skew in the responding of adhd children: a response time distributional approach. Acta Psychol. 104, 167–190. 10.1016/S0001-6918(00)00019-6 10900704

[B13] Luce R. D. (1986). Response Times: Their Role in Inferring Elementary Mental Organization. Oxford psychology series. New York, NY; Oxford: Oxford University Press; Clarendon Press.

[B14] McVay J. C. Kane M. J. (2012). Drifting from slow to “D'oh!”: working memory capacity and mind wandering predict extreme reaction times and executive control errors. J. Exp. Psychol. Learn. Mem. Cogn. 38:525. 10.1037/a0025896 22004270 PMC3395723

[B15] Navarro-Pardo E. Navarro-Prados A. B. Gamermann D. Moret-Tatay C. (2013). Differences between young and old university students on a lexical decision task: evidence through an ex-Gaussian approach. J. Gen. Psychol. 140, 251–268. 10.1080/00221309.2013.817964 24837819

[B16] Ratcliff R. Love J. Thompson C. A. Opfer J. E. (2012). Children are not like older adults: a diffusion model analysis of developmental changes in speeded responses. Child Dev. 83, 367–381. 10.1111/j.1467-8624.2011.01683.x 22188547 PMC3267006

[B17] Ratcliff R. McKoon G. (2008). The diffusion decision model: theory and data for two-choice decision tasks. Neural Comput. 20, 873–922. 10.1162/neco.2008.12-06-420 18085991 PMC2474742

[B18] Sternberg S. (1966). High-speed scanning in human memory. Science 153, 652–654. 10.1126/science.153.3736.652 5939936

[B19] Van Zandt T. (2000). How to fit a response time distribution. Psychon. Bull. Rev. 7, 424–465. 10.3758/BF03214357 11082851

[B20] West R. (1999). Age differences in lapses of intention in the stroop task. J. Gerontol. Ser. B Psychol. Sci. Soc. Sci. 54, P34–P43. 10.1093/geronb/54B.1.P34 9934394

[B21] West R. Alain C. (2000). Age-related decline in inhibitory control contributes to the increased stroop effect observed in older adults. Psychophysiology 37, 179–189. 10.1111/1469-8986.3720179 10731768

[B22] Wickelgren W. A. (1977). Speed-accuracy tradeoff and information processing dynamics. Acta Psychol. 41, 67–85. 10.1016/0001-6918(77)90012-9

