# Corrigendum: ExGUtils: A Python Package for Statistical Analysis With the ex-Gaussian Probability Density

**DOI:** 10.3389/fpsyg.2018.01108

**Published:** 2018-06-26

**Authors:** Carmen Moret-Tatay, Daniel Gamermann, Esperanza Navarro-Pardo, Pedro Fernández de Córdoba Castellá

**Affiliations:** ^1^Department of Neuropsychology, Methodology, Basic and Social Psychology, Faculty of Psychology, Universidad Católica de Valencia San Vicente Mártir, Valencia, Spain; ^2^Instituto de Física, Universidade Federal do Rio Grande do Sul (UFRGS), Porto Alegre, Brazil; ^3^Department of Developmental and Educational Psychology, Faculty of Psychology, Universitat de Valencia, Valencia, Spain; ^4^Grupo de Modelización Interdisciplinar, Instituto Universitario de Matemática Pura y Aplicada, InterTech, Universitat Politècnica de València, Valencia, Spain

**Keywords:** response times, response components, python, ex-Gaussian fit, significance testing

We hereby inform that there was an error in the email of the corresponding author. This is how the author should be contacted: mariacarmen.moret@ucv.es.

The original article has been updated.

## Conflict of interest statement

The authors declare that the research was conducted in the absence of any commercial or financial relationships that could be construed as a potential conflict of interest.

