# Supplementary material for: ExGUtils: A Python Package for Statistical Analysis With the ex-Gaussian Probability Density
Source: Front Psychol. 2018 May 1;9:612. doi: 10.3389/fpsyg.2018.00612 (PMC5939052; doi:10.3389/fpsyg.2018.00612)
Supplement: Supplementary file 1 [file Data_Sheet_1.zip › appendix/appendix.pdf]

ExGUtils: A python package for statistical analysis with the ex-Gaussian probability density

C. Moret-Tatay[a], D. Gamermann[b], E. Navarro-Pardo[c], and P. Fernández de Córdoba[d]

[a]Departamento de Neuropsicobiología, Metodología y Psicología Social - Facultad de Psicología, Magisterio y Ciencias de la Educación, Sede de San Juan Bautista. Universidad Católica de Valencia, San Vicente Mártir - Calle. Guillem de Castro 175, 46008- Valencia, Spain.

[b]Universidade Federal do Rio Grande do Sul (UFRGS) - Instituto de Física, Av. Bento Gonçalves 9500, Porto Alegre, Rio Grande do Sul

[c]Department of Developmental and Educational Psychology - Faculty of Psychology, Universitat de València. Av. Blasco Ibáñez, 21 46010 - Valencia, Spain.

[d]Instituto Universitario de Matemática Pura y Aplicada - IUMPA, Universidad Politécnica de Valencia, E-46022 Valencia, Spain

April 24, 2018

A Python scripts and functions

Here one finds examples of code programed in python using functions from the `ExGUtils` package in order to implement some of the methods discussed in the text.

In listing 1, one finds a quick command line in order to estimate the cutoff point where one expect to find less than 0.1% of a sample obtained from an ex-Gaussian distribution.

In listing 2, two functions are implemented to evaluate, from a data sample, its KS statistic in relation to a known ex-Gaussian distribution.

In listing 3, a function is implemented in order to, from a list containing numerical data, evaluate the probability that a random sample generated from an ex-Gaussian distribution with known parameters, after being fitted to an ex-Gaussian distribution through the maxLKHD method will have a bigger KS statistic than the empirical data. In listing 4 the same function is implemented but the fits are done using the minSQR method.

Listing 1: Determining a cutoff point.

```
>>> from ExGUtils.uts import *
>>> exg_ppf(0.999, 451.09, 47.33, 146.81)
1472.8468996267395
```

Listing 2: Functions to calculate the Kolmogorov-Smirnov statistic.

```
def CDF(data):
    x = list(set(data))
    x.sort()
    y = [data.count(ele) for ele in x]
    y = [sum(y[0:ii])+1 for ii in xrange(len(y))]
    return x, y

def KS_stat(datas, mu, sig, tau, tot):
    x, y = CDF(datas)
    y2 = [tot*exg_cdf(ele, mu, sig, tau) for ele in x]
    N = len(y2)
    diffs = [abs(y[ii]-y2[ii]) for ii in xrange(N)]
    return max(diffs)
```

Listing 3: Function to find the probability p1 (fitting done with maxLKHD).

```
def p1(xi, mu, sig, tau, reps=1000, eps=1.e-10):
    N = len(xi)
    xxx = 1./reps
    KSemp = KS_stat(xi, mu, sig, tau, N)
    pval = 0.
    kss = [0. for ii in xrange(reps)]
    for ii in xrange(reps):
        datas = [exg_rvs(mu, sig, tau) for jj in xrange(N)]
        nmu, nsig, ntau = maxLKHD(datas, eps=eps)
        KSrand = KS_stat(datas, nmu, nsig, ntau, N)
        kss[ii] = KSrand
        if KSrand>KSemp: pval += xxx
    return pval, stats(kss)
```

Listing 4: Function to find the probability p2 (fitting done with minSQR).

```
def p2(xi, mu, sig, tau, reps=1000, eps=1.e-10):
    N = len(xi)
    xxx = 1./reps
    KSemp = KS_stat(xi, mu, sig, tau, N)
    pval = 0.
    kss = [0. for ii in xrange(reps)]
    for ii in xrange(reps):
        datas = [exg_rvs(mu, sig, tau) for jj in xrange(N)]
        [x, y] = histogram(datas, norm=1);
        nmu, nsig, ntau = minSQR(x, y, mu, sig, tau, eps=eps)
        KSrand = KS_stat(datas, nmu, nsig, ntau, N)
        kss[ii] = KSrand
        if KSrand>KSemp: pval += xxx
    return pval, stats(kss)
```

B Experimental Datasets

In [1], two groups of 40 individuals each where presented with two different trial tasks comprised of 90 words in each task. From the 90 words, 30 are considered high frequency, 30 low frequency ones and 30 were pseudowords (text similar to a word, but non existing in a dictionary). The task was to distinguish between the real and the fake words and the response time of each person for each word (or pseudoword) was recorded.

The two groups were formed one by elder people and the second by young university students. The data files provided as supplementary material for download contain the recorded times for each experiment in the different groups (`elder_` for elder people and `young_` for young people), separated by types of words (`_hf` for high frequency, `_lf` for low frequency and `_pseudo` for pseudowords) and experiment set (`_yn` for a yes/no task and `_gng` for a go/no go task). In the files, each line corresponds to a single participant. In the files or tables where a tag is not indicated, it means that all data for the possible tags have been put together. Also note that, in the analysis done in the present manuscript, in order to have enough statistic, the analysis has not been done in a per participant basis, but all participants in a same dataset have been pooled together for illustrative proposes only [2].

The data also indicates if the participant made a mistake in a given word by reporting the response time as negative. In the analysis performed here all negative numbers have been removed from the datasets.

References

[1] E. Navarro-Pardo, A. B. Navarro-Prados, D. Gamermann, and C. Moret-Tatay, "Differences between young and old university students on a lexical decision task: evidence through an ex-Gaussian approach," *J Gen Psychol*, vol. 140, no. 4, pp. 251–268, 2013.

[2] D. Cousineau, J.-P. Thivierge, B. Harding, and Y. Lacouture, "Constructing a group distribution from individual distributions.," *Canadian Journal of Experimental Psychology/Revue canadienne de psychologie expérimentale*, vol. 70, no. 3, p. 253, 2016.
